# Glucose hypometabolism in the Auditory Pathway in Age Related Hearing Loss in the ADNI cohort

**DOI:** 10.1016/j.nicl.2021.102823

**Published:** 2021-09-21

**Authors:** Fatin N. Zainul Abidin, Marzia A. Scelsi, Sally J. Dawson, Andre Altmann, for the, Alzheimer's Disease Neuroimaging Initiative

**Affiliations:** a UCL Ear Institute, University College London, London, UK; b Centre for Medical Image Computing, Department of Medical Physics and Biomedical Engineering, University College London, London, UK

**Keywords:** Hearing loss, 18F-FDG PET, Volume of interest analysis, Genome-wide association study Auditory cortex, Heschl’s gyrus, Neuroimaging genetics, Inferior colliculus, ADNI

## Abstract

**Purpose:**

Hearing loss (HL) is one of the most common age-related diseases. Here, we investigate the central auditory correlates of HL in people with normal cognition and mild cognitive impairment (MCI) and test their association with genetic markers with the aim of revealing pathogenic mechanisms.

**Methods:**

Brain glucose metabolism based on FDG-PET, self-reported HL status, and genetic data were obtained from the Alzheimer’s Disease Neuroimaging Initiative (ADNI) cohort. FDG-PET data was analysed from 742 control subjects (non-HL with normal cognition or MCI) and 162 cases (HL with normal cognition or MCI) with age ranges of 72.2 ± 7.1 and 77.4 ± 6.4, respectively. Voxel-wise statistics of FDG uptake differences between cases and controls were computed using the generalised linear model in SPM12. An additional 1515 FDG-PET scans of 618 participants were analysed using linear mixed effect models to assess longitudinal HL effects. Furthermore, a quantitative trait genome-wide association study (GWAS) was conducted on the glucose uptake within regions of interest (ROIs), which were defined by the voxel-wise comparison, using genotyping data with 5,082,878 variants available for HL cases and HL controls (N = 817).

**Results:**

The HL group exhibited hypometabolism in the bilateral Heschl’s gyrus (k_left_ = 323; k_right_ = 151; T_left_ = 4.55; T_right_ = 4.14; peak P_uncorr_ < 0.001), the inferior colliculus (k = 219;T = 3.53; peak P_uncorr_ < 0.001) and cochlear nucleus (k = 18;T = 3.55; peak P_uncorr_ < 0.001) after age correction and using a cluster forming height threshold P < 0.005 (FWE-uncorrected). Moreover, in an age-matched subset, the cluster comprising the left Heschl’s gyrus survived the FWE-correction (k_left_ = 1903; T_left_ = 4.39; cluster P_FWE-corr_ = 0.001). The quantitative trait GWAS identified no genome-wide significant locus in the three HL ROIs. However, various loci were associated at the suggestive threshold (p < 1e-05).

**Conclusion:**

Compared to the non-HL group, glucose metabolism in the HL group was lower in the auditory cortex, the inferior colliculus, and the cochlear nucleus although the effect sizes were small. The GWAS identified candidate genes that might influence FDG uptake in these regions. However, the specific biological pathway(s) underlying the role of these genes in FDG-hypometabolism in the auditory pathway requires further investigation.

## Highlights

• The voxel-wise comparison between older adults with hearing loss and without hearing loss revealed FDG hypometabolism in bilateral Heschl’s gyrus.
• Additional FDG hypometabolism in the inferior colliculus and cochlear nucleus was localized after age-adjustment.
• Decline in FDG metabolism in the cochlear nucleus was accelerated in people with hearing loss.
• Various genetic loci demonstrated suggestive associations with glucose metabolism in hearing loss-associated brain regions.

## 1 Introduction

Hearing loss affects millions of people around the world and The Global Burden of Disease Study found that hearing loss is the fourth leading cause of disability globally (Cunningham and Tucci, 2017, World Health Organization, 2018). The incidence and prevalence of hearing loss increases with age (Yueh et al., 2003, Cruickshanks et al., 1998, Lin et al. 2011). Presbycusis, or age-related hearing loss (ARHL), is the most prevalent form of hearing loss which affects 80% of adults over the age of 70 years (Mares-perlman and Nondahl, 1998). It is characterised by reduced bilateral hearing sensitivity, impaired localization of sound sources, decreased ability to understand speech in background noise, and slowed central processing of acoustic input (Gates and Mills, 2005). It is caused by dysfunction in the transduction of sound-induced vibrations into electrical signals by sensory hair cells in the cochlea and central nervous system dysfunction in auditory signal pathway although the respective contribution of central and peripheral pathologies remain unclear (Shen, 2018, Liberman, 2017). Treatments include auditory training (Ferguson and Henshaw, 2015), hearing aids and cochlear implants for severe hearing loss. Untreated hearing impairment contributes to social isolation, depression, and is a risk factor for dementia (Lin et al., 2011). In midlife, hearing loss is the greatest modifiable risk factor for dementia, alongside hypertension and obesity early intervention might help in delaying or reducing the risk of developing dementia in later life (Livingston et al., 2020).

Age-related hearing loss is currently attributed to the decline of the peripheral auditory system or deficits in the processing of auditory signals along the central auditory nervous system. The latter cause can be investigated using brain imaging methods such as functional magnetic resonance imaging (fMRI) or fluorodeoxyglucose (^18^F) positron emission tomography (FDG) positron emission tomography (PET) imaging. However, PET is usually preferred over fMRI in functional auditory cortex studies of hearing as PET is a passive imaging technique that does not produce background noise (Talavage et al., 2014). Noise during fMRI acquisition can interfere with the results and lead to false positive associations, however, specialized pulse sequences can be used to mitigate this effect (Talavage et al., 1999). The few existing neuroimaging studies focusing on hearing-related phenotypes typically involve a small number of participants (<50), and have suggested that primary auditory cortices are affected (Verger et al., 2017, Griffiths et al., 2001, Eckert et al., 2012, Boyen et al., 2013, Speck et al., 2020, Wang et al. 2016). Sound processing begins in the cochlea itself, then via the auditory nerve to the cochlear nuclei and continues up to the inferior colliculi, the medial geniculate bodies and finally the auditory cortex (Griffiths et al., 2001, Cooper, 1948). Lack of stimulation resulting from dysfunction in the inner ear as well as the effect of ageing is likely to cause changes in brain grey matter density in the auditory cortex (Ouda et al., 2015). However, hearing loss-related changes in brain structures along auditory pathways have yet to be discovered in a neuroimaging study. Multiple factors such as genetics, brain structure and function contribute to auditory processing as well as auditory problems such as ARHL and their individual roles are challenging to untangle. One recent genetics study discovered 44 genomic loci for self-reported adult hearing difficulty (Wells et al., 2019). Thus, using neuroimaging and imaging genetics to investigate effect of genetic variation on brain function may provide novel insights on the pathological processes underlying age-related hearing loss.

To date, only a few imaging studies that deal with normal hearing (Griffiths et al., 2001) and hearing-related problems have been undertaken and these have largely used small samples (Verger et al., 2017, Eckert et al., 2012, Boyen et al., 2013, Speck et al., 2020, Wang et al. 2016). An FDG-PET imaging study with 27 late-onset deafness participants and matched controls found only one cluster with reduced metabolism: the right associative auditory cortex, and increased metabolism within distant brain areasareas (Chaudat and Felician, 2017). An MRI study of 49 older adults found reduced grey matter volume in the ﻿auditory cortex to be associated with high frequency hearing loss (Eckert et al., 2012). A study using voxel-based analyses to discriminate between tinnitus and hearing loss found no differences in grey matter volumes (Boyen et al., 2013). Reduced as well as increased grey matter volume in the primary auditory cortex and reduced glucose uptake in the inferior colliculus (IC) and primary auditory cortex were observed using MRI and PET imaging on 42 and 13 unilateral hearing loss subjects, respectively (Speck et al., 2020, Wang et al. 2016). Other FDG-PET studies have been undertaken in younger adults with early onset hearing loss with different aims (Speck et al., 2020, Han et al., 2019, Lee et al. 2003). All these studies found that auditory cortices are affected in hearing loss, however, they were not able to capture the complete auditory processing pathway probably due to lack of power from limited sample sizes.

Here, we conduct the largest functional neuroimaging study to investigate glucose metabolism differences in ARHL compared to healthy subjects at rest and to investigate the central auditory correlates of hearing loss in aged adults. To this end we leverage FDG-PET imaging scans from >1,000 subjects collected as part of the Alzheimer’s Disease Neuroimaging Initiative (ADNI) (Jagust et al. 2010). Further, using these data we present the results of the first imaging genetics analysis of ARHL as well as investigating the longitudinal decline in glucose metabolism in ARHL. We hypothesize that brain regions concerned with auditory processing, such as the primary auditory cortex, would show reduced glucose metabolism in subjects with ARHL. Our findings contribute to a better understanding of the genetic influences of hearing loss as they effect central auditory function, a pre-requisite for the development of new prevention and treatment strategies.

## 2 Materials and methods

Data used in this study were obtained from the Alzheimer’s Disease Neuroimaging Initiative (ADNI) database (adni.loni.usc.edu) (Jagust et al. 2010). Data used in the preparation of this article were obtained from the Alzheimer’s Disease Neuroimaging Initiative (ADNI) database (adni.loni.usc.edu). The ADNI was launched in 2003 as a public–private partnership, led by Principal Investigator Michael W. Weiner, MD. The primary goal of ADNI has been to test whether serial magnetic resonance imaging (MRI), positron emission tomography (PET), other biological markers, and clinical and neuropsychological assessment can be combined to measure the progression of mild cognitive impairment (MCI) and early Alzheimer’s disease (AD). The participants are adults aged 55–90 years and data in ADNI database are labelled as cognitively normal, MCI, or AD.

### 2.1 Participants

All data including the baseline demographic characteristics, medical history, physical examination, and neurological examination were downloaded from ADNI database. In this study we only investigated subjects diagnosed as healthy cognition (HC) or MCI at their initial visit. Participants with AD were excluded from this study due to the heterogenous patterns of brain atrophy in AD (Rassmussen et al., 2012). Participants with HL were defined as having specific terms related to HL registered/reported in either physical examination, medical history, and neurological examination as previously defined in a study on hearing loss in ADNI cohort (Xu et al., 2019). The search terms for screening for HL include “hear”, “auditory”, “ear”, “deaf”, “presbycusis” at baseline. Patients with deafness onset during birth/childhood or because of exposure to noise in war/working environment were excluded to maintain homogeneity of the study population. Participants with FDG-PET and MRI images available at baseline or one-year follow-up were considered.

### 2.2 FDG PET acquisition and processing

At the time of this study, 1003 pre-processed ^18^F-FDG PET scans at baseline for HL and control subjects were available and downloaded from ADNI database (adni.loni.usc.edu) (date accessed 2/05/2019). Data pre-processing steps and details are accessible online (http://adni.loni.usc.edu/data-samples/data-types/) and described in detail elsewhere (Jagust et al. 2010). Briefly, 185 MBq of [^18^F]-FDG was injected intravenously. Six 5-min frames were acquired 30 min post-injection. Each frame of a given baseline image series was co-registered to the first acquired frame and the image series was combined into a dynamic image set. The image set was then averaged, reoriented to a standard space (voxel grid size 160 × 160 × 96, voxel size 1.5 × 1.5 × 1.5 mm^3^), intensity normalized, and smoothed with an 8 mm FWHM kernel.

A total of 1003 pre-processed T_1_-weighted magnetic resonance scans were downloaded for the same subjects at the baseline. Details about the magnetic resonance pre-processing can be found in Jack Jr et al. (2008). PET images in nifti format were co-registered to their corresponding MRI T_1_-weighted image using normalized mutual information in SPM12 (Friston et al. 1994). The T_1_-MRI images were registered to Montreal Neurological Institute (MNI) space and transformation was then applied to the co-registered PET images using SPM12, thus providing PET images in MNI space. PET-T_1_ registrations were visually assessed to ensure correct alignment. In order to remove inter-individual variability in tracer metabolism, standardized uptake value ratio (SUVR) was computed. To this end FDG-PET voxel intensity was divided by the intensity in a joint pons and vermis ROIs (Rassmussen et al., 2012), due to their preserved glucose metabolism in AD and MCI. These SUVRs were then used as normalized measurement of voxel-level cortical glucose uptake for each subject.

### 2.3 Statistical analysis

The backbone of the statistical analysis is the voxel-wise comparison of FDG SUVR between HL subjects and controls using the two-sample *t*-test framework of the generalized linear model in SPM12. All tested models were adjusted for sex and education. Moreover, age (at PET imaging) represents a potential main confounder for investigating age-related HL in this cohort. Correctly accounting for age as a confounder when investigating age-related disorders is a challenge. In order to avoid only picking up age-related effects on glucose metabolism, we followed three strategies to address this: (1) age as covariate, (2) matching case and control group on age, and (3) adjusting voxel-level SUVR for age prior to the SPM analysis. For the latter strategy we followed the methodology introduced by Dukart et al. (2011). Briefly, GLMs were calculated for all SUVR voxels$y_{c}$ separately across the set of control subjects (non-HL), *c* using only age as the explanatory variable, $X_{c}$ :

(1) $$y_{c}=X_{c}\beta +\varepsilon_{c}$$

Regression coefficients $\beta_{c}$ from the voxel-wise linear regressions were extracted providing a voxel-wise map of the effect of age on SUVR. Corrected SUVR values $y_{\mathrm{corrected}}$ were calculated for every voxel in each participant (controls and HL cases) by subtracting the expected effect of participant age ($\beta_{c}X_{A}$) from the participant voxel SUVR value ($y_{A}$):

(2) $$y_{\mathrm{corrected}}=y_{A}-\beta_{c}X_{A}$$

We report uncorrected as well as FWE-corrected p-values (based on random field theory) for peaks and clusters. Furthermore, SPM statistical analysis results are reported in an image highlighting the significant set of voxels and accompanied by the list of clusters and their statistical significance. For follow-up analyses, ROIs associated with HL were defined as clusters of voxels with cluster-level uncorrected p-value < 0.005 as cut-off for cluster building. The average SUVR in each of these ROIs was later used for genetic analysis (see below). In a complementary analysis to investigate the effect of normal aging on cortical glucose uptake, a voxel-level map of beta values $\beta_{c}$ resulting from method (3) were extracted and later visualised together with hearing loss effects to illustrate the aging effects on glucose uptakes.

### 2.4 Longitudinal analysis

In order to test whether ARHL leads to a faster decline in glucose metabolism in the ROIs associated with HL, we leverage the available longitudinal FDG-PET imaging data within ADNI. Briefly, a total of 1515 subsequent PET scans for 618 of the 1003 subjects were extracted and registered to the baseline FDG-PET scan, which was already registered to MNI space as described above. Average glucose metabolism was computed for the HL ROIs, which were identified in the voxel-wise cross-sectional analysis, and intensity normalized to the joint pons-vermis ROI. A linear mixed effect model with random intercept and random slopes for time since baseline was used to analyse the timeseries data. The target was the SUVR in the HL ROI and fixed effects were age at baseline, sex, cognitive diagnosis, MMSE, ARHL status and time since baseline. We tested for an interaction between time since baseline and ARHL status on regional glucose metabolism.

### 2.5 Genetic data acquisition and processing

﻿Single nucleotide polymorphism (SNP) genotyping data is available for n = 1674 subjects across all ADNI phases. The 1674 samples were genotyped on three Illumina platforms: ﻿Human610-Quad, HumanOmniExpress and Omni 2.5 M. Details on data processing quality control and imputation have been described previously (Scelsi, et al., 2018). Briefly, QC steps were conducted at subject and SNP level. At subject level, call rate at 10% and concordance between chip-inferred and self-reported sex were performed. At SNP level, standard QC steps were conducted to ensure consistency with the Haplotype Reference Consortium (release 1.1) reference panel used for imputation. These included strand consistency, allele names, position, reference/alternative allele assignments and minor allele frequency (MAF) deviations from the reference panel following QC steps. The imputation was performed using the Sanger Imputation Server ﻿(https://imputation.sanger.ac.uk/) with SHAPEIT for phasing (Delaneau et al., 2013) and the entire Haplotype Reference Consortium (release 1.1) reference panel (McCarthy et al. 2016) on data from the three platforms separately. Genotypes were hard-called using a threshold of > 0.9. Next, genotypes from the three platforms were merged and SNPs with MAF > 5% and genotyping rate > 0.9 were retained. Finally, subjects with predicted Central European ancestry of 80% or more (determined using SNPweights (Chen et al. 2013) were retained; the relatedness matrix between subjects was computed using the remaining autosomal SNPs and the dataset was trimmed to remove subjects with relatedness > 0.1. For the remaining subjects the first five PCA components were computed in PLINK v1.9 (Chang et al., 2015) and were used to account for population structure in the genome-wide analyses. Overall, 5,082,878 variants were available for genotyping data in cases and controls.

### 2.6 Association analysis

Genetic data was available for a subset of participants (815 out of 1003) with imaging data (Table 1). Genome-wide quantitative analysis was performed on all subjects in our hearing loss study for whom genome-wide data was available. As traits for the quantitative analysis, we used the average glucose uptake in regions of interest identified from the imaging statistical analysis. We used rank-based inverse normal transformation, implemented in the R package RankNorm (v.1.0.0), to ensure a gaussian distribution of the target phenotypes and to minimize the effect of outliers. We fitted additive genetic models using PLINK v1.9 (www.cog-genomics.org/plink/1.9/) (Chang et al., 2015) linear regression on averaged and normalised voxel intensities within ROIs; models were adjusted for age, sex, years of education, three principal components of population structure, cognitive diagnosis and HL status. In the resulting summary statistics, we regarded genetic variants with p-value < 5e-08 as genome-wide significant and p-value < 1e-05 as suggestive. The SNP2GENE function of the FUMA web tool (Watanabe et al., 2017) was used to identify independent signals within highly associated regions and to map them to the nearest gene, as well as to generate Manhattan, QQ and regional association plots. To access and control for population stratification, we examined the QQ-plots and genomic control inflation factors for all GWASs.

**Table 1:** Subgroup characteristics for subjects with HL and non-HL. Mean ± standard deviation, ARHL age-related hearing loss, MCI mild cognitive impairment, HC healthy cognition, MMSE Mini Mental State Examination, GI genetic information. * Included in the genetic study.

	HL	Non-HL	T-test (t, p)	Chi^2^ test (χ^2^, df, p)
Number	261	742	n/a	n/a
Male/Female	190/71	379/363	n/a	36.22, 1, 1.759e-09
Age (years)	77.4 (6.4)	72.2 (7.1)	10.96, <2.2e-16	n/a
Age range (min-max)	56.5 - 91.5	55.1 - 89.1	n/a	n/a
Years of Education	16.3 (2.8)	16.0 (2.7)	1.16, 0.246	n/a
Smoking (yes/no)	111/80	276/209	n/a	0.04, 1, 0.842
Diabetes (yes/no)	23/230	65/631	n/a	1.08e-30, 1, 1.0
Hypertension (yes/no)	124/129	331/365	n/a	0.10, 1, 0.747
APOE Ɛ4 (1/2/3)	163/82/16	415/256/65	n/a	3.59, 1, 0.166
MCI/HC diagnosis	174/87	526/216	n/a	1.44, 1, 0.230
MMSE (score)	27.9 (1.7)	28.2 (1.7)	-2.0, 0.046	n/a
GI available*	228	587	n/a	n/a

### 2.7 Genetic correlation analyses

A cross-trait LD Score regression method (Bulik-Sullivan et al. 2015) was used to evaluate the genome-wide genetic correlation between glucose intakes in region of interest GWAS from this imaging study, hearing difficulty with background noise GWAS and hearing aid GWAS. Unlike Mendelian randomization, which simply employs significantly associated SNPs, cross-trait LD Score regression makes use of the effects of all SNPs to estimate the correlation.

## 3 Results

Table 1 shows subgroup characteristics for HL and non-HL subjects. A total of 1003 participants which include 261 cases of HL and 742 controls/non-HL were studied. Age at imaging was significantly different between HL and non-HL groups, with non-HL participants being on average 5.2 years younger (p-value < 2.2e-16). Male to female proportion was also different between HL and non-HL with higher prevalence of HL among males (χ^2^ = 36.224, p-value = 1.759e-09). Furthermore, we observed slightly lower (on average −0.3) Mini Mental State Examination (MMSE) score in HL than in non-HL (p-value = 0.047). On the other hand, the ratio of HC and MCI diagnoses, smoking, diabetes, hypertension, APOE Ɛ4 status between HL and non-HL did not show any difference (Table 1) with p-values > 0.05, likewise, years of education was not different between these groups. Cognitive status of subjects in our study within non-HL comprised of 71% with MCI and 29% healthy cognition, whereas in HL, 67% of subjects had MCI and 33% were HC.

**Fig. 1:**
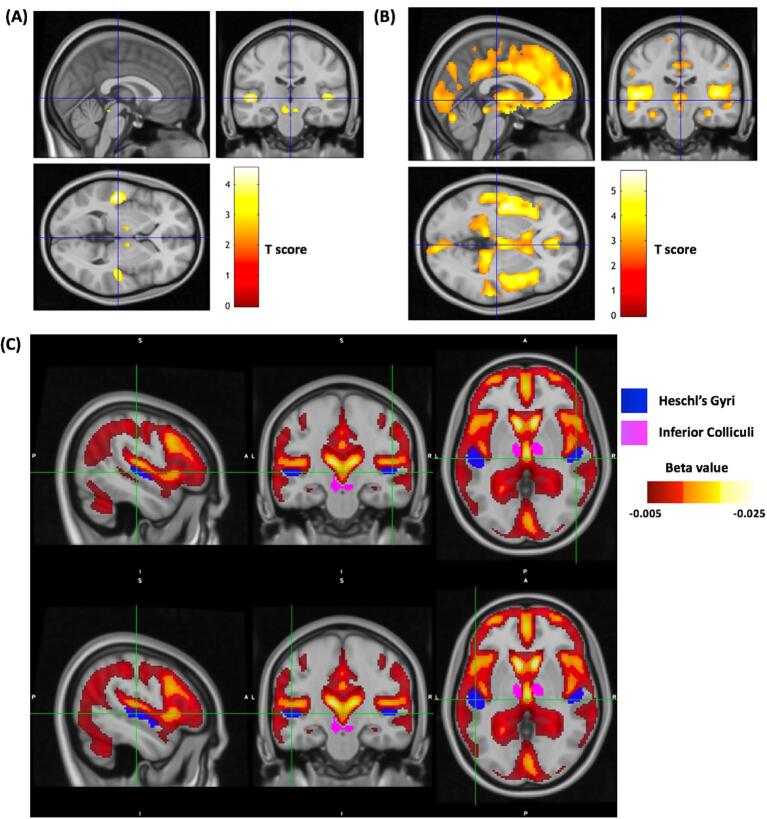
Effects of hearing loss on glucose metabolism. Glucose hypometabolism patterns in bilateral Heschl’s Gyrus and midbrain auditory pathway (e.g., inferior colliculus) are apparent in **(A)** with age correction and **(B)** without age correction at a cluster-forming height threshold p-value = 0.005 (uncorrected) with sagittal, axial and coronal sections at x  = 0, y = 22, z = 1. Colour scale gives Student's *t* statistic for the comparison between HL and non-HL. Years of education and sex are included as covariates in SPM two-sample *t*-test for both. **(C)** shows comparison of effect of aging and the regions of interest selected for GWAS for right hemisphere (top) and left hemisphere (bottom). Regions of interest include bilateral Heschl’s gyrus in blue and inferior colliculus in magenta with sagittal, axial and coronal sections at x = ±50, y = ±20, z = 2. Colour scale gives beta coefficient (*β_c_*) for age in linear regression model for glucose uptake SUVR in all subjects. (For interpretation of the references to colour in this figure legend, the reader is referred to the web version of this article.)

Due to a significant difference in age between HL and non-HL groups, the voxel-wise comparison was done by adjusting for age using formulas (1) and (2). This resulted in four relevant clusters (Table 2**;** Fig. S2; cluster forming height threshold p-value = 0.005 uncorrected) at peak voxel-wise threshold p-value < 0.001 (uncorrected). These clusters formed the region of interest for follow-up analyses: ROIs 1 to 4 correspond to left Heschl’s gyrus (HG), right Heschl’s gyrus, inferior colliculus (IC) and cochlear nucleus (CN), respectively. The peak p-value for the left Heschl’s gyrus (p-value < 0.001, T = 4.55) missed the t-value cutoff for FWE-corrected brain-wide significance T = 4.8. Overall, Heschl’s gyri, inferior colliculus and cochlear nuclei (Fig. 1**A)** were affected by reduced glucose uptake at in HL compared to non-HL, followed by medial geniculate body (Fig. **S3).** Similar results were observed when employing alternative age-correction strategies in method (1), i.e., age as covariate (Fig. **S4**) and (2) matching HL and control group on age (Fig. **S5**), where the cluster comprising the left Heschl’s gyrus survived the FWE-correction (cluster P_FWE-corr_ = 0.001).

**Table 2:** Group comparison between HL and non-HL. Data are from participants with HL compared to controls with adjustment of age prior to test and further corrected for sex, and years of education. L, left hemisphere; R, right hemisphere; P_uncorr_, cluster-level and peak-level uncorrected voxel-wise p-value.

	Cluster-level		Peak-level	MNI coordinate				Region
	P_uncorr_	size	P_uncorr_	X	Y	Z		
ROI 1	0.029	323	<0.001	−48	−24	2	0.144	L Heschl’s gyrus (Griffiths et al., 2001) (Norman-haignere et al., 2013)
ROI 2	0.120	151	<0.001	46	−24	4	0.131	R Heschl’s gyrus (Griffiths et al., 2001, Norman-haignere et al., 2013)
ROI 3	0.066	219	<0.001	−4	–32	−14	0.112	Inferior colliculi (Griffiths et al., 2001)
ROI 4	0.598	18	<0.001	10	−36	−38	0.112	R cochlear nucleus (Griffiths et al., 2001)

To explore the effect of aging further, we investigated the effect of age correction on this result. The voxel-wise comparison between HL and non-HL subjects without adjusting for age revealed very large clusters (ranging from 1,000 – 15,900 voxels) surviving FWE-correction at p-value < 0.05 (Fig. 1**B;** Fig. **S1;** cluster forming height threshold p-value = 0.005 uncorrected) and aging effects were not specific to the auditory cortex (Fig. 1**C**). Regions related to auditory processing, Heschl’s gyrus and inferior colliculus, did not overlap substantially with regions showing age related effects in glucose metabolism.

To rule out a driving effect of cognitive impairment on the results, we conducted a sensitivity analysis where the subjects were restricted to HC and early MCI diagnosis (as provided by ADNI). The results confirmed bilateral hypometabolism in HG as well as hypometabolism in subcortical ROIs (Fig. **S6**).

### 3.1 ARHL did not consistently accelerate decline in glucose metabolism

A total of 618 of the 1003 subjects with HC or MCI had more than one PET scan in the ADNI database. The number of follow-up scans ranged from one to nine, with an average of 2.4 follow-up scans. There were 158 and 460 participants with and without ARHL, respectively. In the linear mixed effect model the HG (beta = 0.006653; t = 1.267, p-value = 0.21) and IC (beta = −0.0016414; t = −0.517; p-value = 0.6) ROIs did not show accelerated decline in subjects with ARHL. However, the CN ROI showed a nominally significantly accelerated decline in glucose metabolism (beta = -0.004067; t = −2.312; p-value = 0.021).

### 3.2 GWAS of regional glucose metabolism

We used the four ROIs identified after age adjustment in the imaging analysis (Table 2) for the genome-wide association analysis on all 815 participants with available genotyping and imaging data (Table1). ROIs 1 and 2 representing the left and right Heschl’s gyri, respectively, were combined into a bilateral ROI: HG; ROI 3 representing the bilateral inferior colliculus (IC) was used as is for GWAS. ROI 4 representing the Cochlear nucleus (CN) was excluded from the post-GWAS analysis due to small cluster size and resulting unreliable statistics. Three independent GWAS were conducted by testing each genetic variant (5,082,878 available SNPs) for an association with HG, IC and CN respectively. Any systematic biases that may present in the association results were investigated by calculating the genomic inflation factor, also known as lambda gc ($\lambda_{\mathrm{gc}}$). We did not observe evidence for widespread inflation in both HG and IC GWAS with $\lambda_{\mathrm{gc}}$ of 0.999, 0.994 and 1.001, respectively, in all samples (Fig. **S7**).

**Fig. 2:**
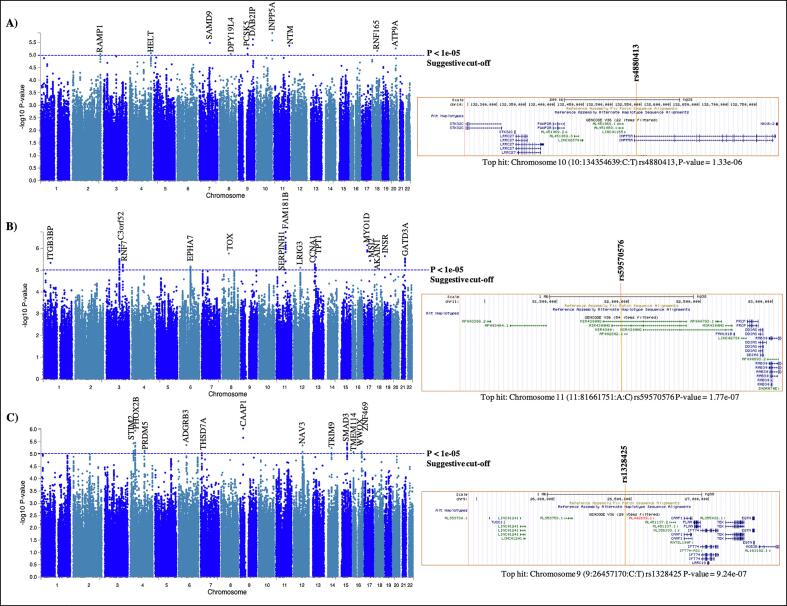
Genome-wide associations with metabolism in HL-related ROIs. Manhattan plots displaying GWAS results for **(A)** bilateral Heschl’s gyri/ROI 1&2 GWAS and **(B)** inferior colliculus/ROI 3 GWAS **(C)** right cochlear nucleus/ROI 4 GWAS of averaged SUVRs within ROIs for all subjects with genetics data available. The Manhattan plots display the -log_10_ P-values of all SNPs tested for association with glucose uptake in ROIs. The threshold for genome-wide suggestive threshold (p-value < 1e-05) is indicated by a blue horizontal solid line. Loci that reached genome-wide suggestive level are annotated with gene symbol. Each Manhattan plot accompanied by the location of the top SNP with surrounding genes. (For interpretation of the references to colour in this figure legend, the reader is referred to the web version of this article.)

From the GWAS analyses, we did not identify any genome-wide significant association signals (p-value < 5e-08), but there are some loci harboring genome-wide suggestive SNPs (p-value < 1e-05) in bilateral Heschl’s gyrus GWAS and inferior colliculus GWAS. Each of these SNPs was mapped to the nearest gene (Table 3). The top SNP from Heschl’s gyrus GWAS was mapped to the *INPP5A* protein coding gene (rs4880413, chr10:134354639, intron of *INPP5A*, p-value = 1.33e-06)**.** The top SNP from inferior colliculus GWAS was mapped to the *FAM181B* protein coding gene (rs59570576, chr9: 124391684, downstream of *FAM181B*, p-value = 1.77e-07) and also located within *MIR4300HG* long non-coding RNA gene (Fig. 2**B**). Additional suggestive SNPs for HG, IC, and CN GWAS are listed in Fig. 2 and Table 3**.** Overall, 10, 15 and 13 independent loci were identified in HG, IC and CN GWAS at the suggestive level, respectively (Fig. 2 and Table 3). In addition, we investigated whether there was any shared genetic architecture between HG from our GWAS analysis and self-reported hearing difficulty and hearing aid phenotypes from Wells et al. (2019). We found no significant genetic correlation (rg) for HG glucose metabolism with hearing difficulty (rg = -0.055, p-value = 0.641) and with hearing aid use (rg = -0.047, p-value = 0.679) and thus, no inferable genetic architecture overlap between our hearing loss neuroimaging genetic phenotype and hearing difficulty with background noise and hearing aid phenotypes by Wells et al. (2019) could be identified.

**Table 3:** Summary statistics for lead SNPs that reached a suggestive threshold of p-value < 1e-05, ordered by chromosome for bilateral Heschl’s gyrus, inferior colliculus and cochlear nucleus GWAS. Chr, chromosome; bp, base position; SNP, lead SNP; refA, reference allele; altA, alternative allele; beta, effect size; se, standard error of the beta; P, association test p-value.

**Chr**	**bp**	**SNP**	**altA**	**refA**	**beta**	**se**	**P**	**Nearest gene**
**Bilateral Heschl’s gyrus GWAS**								
10	134,354,639	rs4880413	C	T	0.267	0.048	1.33e-06	*INPP5A*
9	124,391,684	rs78424955	A	G	0.315	0.066	2.38e-06	*DAB2IP*
7	92,687,498	rs11980100	A	C	−0.308	0.065	3.30e-06	*SAMD9*
11	131,816,630	rs511311	A	G	0.225	0.048	4.25e-06	*NTM*
9	78,581,102	rs17061846	C	G	0.341	0.074	5.48e-06	*PCSK5*
20	50,201,728	rs73133002	C	T	−0.494	0.108	5.50e-06	*ATP9A*
18	44,040,660	rs17766830	C	T	0.239	0.052	6.83e-06	*RNF165*
4	185,874,623	rs12502026	A	G	−0.237	0.052	8.50e-06	*HELT*
2	238,822,161	rs75026491	A	C	−0.279	0.062	9.06e-06	*RAMP1*
8	95,736,479	rs28523271	A	G	−0.475	0.106	9.10e-06	*DPY19L4*
**Inferior colliculus GWAS**								
11	81,661,751	rs59570576	A	C	−0.280	0.053	1.77e-07	*FAM181B*
3	111,819,524	rs12053863	A	C	−0.255	0.051	7.10e-07	*C3orf52*
17	30,953,776	rs321157	A	G	−0.255	0.051	7.19e-07	*MYO1D*
8	59,934,166	rs10957077	A	G	−0.247	0.051	1.73e-06	*TOX*
19	7,101,673	rs35207600	C	T	−0.521	0.109	2.30e-06	*INSR*
13	45,905,074	rs11618819	G	T	0.437	0.092	2.49e-06	*TPT1*
21	45,617,328	rs2032327	A	G	0.231	0.049	2.88e-06	*GATD3A*
17	55,731,466	rs17762121	A	G	−0.279	0.059	3.71e-06	*MSI2*
1	63,941,202	rs6588035	A	G	−0.258	0.055	4.61e-06	*ITGB3BP*
13	37,038,562	rs73534366	C	T	0.262	0.057	5.40e-06	*CCNA1*
3	141,407,056	rs6798834	A	G	−0.221	0.048	5.61e-06	*RNF7*
6	94,679,686	rs1352378	G	T	−0.223	0.049	6.79e-06	*EPHA7*
12	59,179,375	rs2124517	C	T	−0.229	0.051	7.70e-06	*LRIG3*
11	75,277,628	rs584961	A	G	−0.010	0.080	9.51e-06	*SERPINH1*
18	5,099,428	rs60475118	A	G	−0.230	0.051	9.74e-06	*AKAIN1*
**Cochlear nucleus GWAS**								
9	26,457,170	rs1328425	C	T	0.23	0.047	9.24e-07	*CAAP1*
4	41,793,147	rs6851412	C	T	−0.243	0.049	1.12e-06	*PHOX2B*
16	88,453,759	rs4782515	C	T	−0.250	0.053	3.20e-06	*ZNF469*
15	67,460,009	rs11638064	A	G	−0.264	0.056	3.58e-06	*SMAD3*
4	27,739,484	rs990966	A	C	−0.106	0.052	3.64e-06	*STIM2*
6	68,578,367	rs59067831	G	T	0.532	0.115	4.29e-06	*ADGRB3*
12	78,288,587	rs2045989	C	T	0.243	0.052	4.44e-06	*NAV3*
4	41,744,499	rs73139112	A	G	−0.316	0.068	5.02e-06	*PHOX2B*
14	51,568,946	rs12589468	A	G	0.229	0.050	5.70e-06	*TRIM9*
4	121,479,398	rs6856719	A	G	−0.414	0.091	7.27e-06	*PRDM5*
16	78,857,425	rs56209917	A	G	0.298	0.066	8.03e-06	*WWOX*
16	8,607,848	rs74007850	C	T	0.374	0.083	8.28e-06	*TMEM114*
7	11,619,598	rs2189639	C	T	−0.243	0.054	9.30e-06	*THSD7A*

## 4 Discussion

Glucose metabolism of older adults with hearing loss was lower compared to controls in regions of the auditory pathway, including bilateral Heschl’s gyri and inferior colliculus and right hemisphere cochlear nucleus. The brain regions primarily involved in auditory processing such as the Heschl’s gyri, inferior colliculus, cochlear nucleus and medial geniculate bodies showed the strongest associations with HL (Fig. **S3**), but below the brain-wide FWE-corrected significance threshold. Of note, an analysis focusing *a priori* on the bilateral primary auditory cortices using an appropriate mask, led to a cluster in the left HG surviving the FWE correction (FWE-corr p-value = 0.039). To the best of our knowledge, this is the first demonstration in a cross-sectional study that hypometabolism of glucose is observed in both primary auditory cortex and brain stem nuclei of ARHL participants using FDG-PET scans; with 1003 participants it is the largest study of its kind so far.

In this neuroimaging study, we also attempted to dissociate the effect of normal aging from hearing loss. Hearing loss is common in older adults of over 65 years of age, and in this secondary data analysis the two groups of interest, i.e., older adults with and without hearing loss, exhibited a significant age difference. Applying the age-correction using aging effects learned in non-HL subjects substantially improved detection of disease related FDG metabolism in HL (Fig. 1**A).** Thus, glucose uptake of structures specific to the auditory pathway appeared to be affected. Age-correction eliminated the wide-spread detected clusters (Fig. 1**B**). The age difference between the HL and non-HL groups likely influenced the exact delineation of the HL effect. In principle, the spatial pattern in this analysis still resembles that of our main result (Fig. 1**A)** once a more stringent cut-off is applied. Moreover, without age-correction (Fig. 1**B)**, the HL effect and general aging effects become difficult to discriminate. Hence, during cluster generation the HL-effect clusters get merged with the aging effect clusters emphasizing the need for adequate age adjustment in this cohort. Overall, the effects of normal aging (Fig. 1**C**) appear not to be specific to the auditory cortex.

The longitudinal component of this study investigated whether HL diagnosis at baseline accelerates the decline in glucose metabolism. The results were inconsistent in that only one of the three ROIs showed a nominally significantly accelerated decline in people with HL (p-value = 0.021). However, longitudinal studies with PET imaging are challenging owing to issues with registration and intensity normalization and thus may have masked any effect in the other two ROIs. In addition, lack of HL-diagnosis at follow-up visits in the ADNI cohort may further obscure the effect of HL on metabolic decline.

The sample size of GWAS has been constantly increasing over the last years in order to increase the statistical power to detect small effect sizes and reach genome-wide significance (p-value < 5e-08). For a GWAS, our imaging genetics is rather small, thus, due to the lack of genome-wide significant findings, we discuss loci at the suggestive level (p-value < 1e-05). In this study, we found that the rs4880413 variant (Fig. 2**A**) within intron of the Inositol polyphosphate-5-phosphatase A (*INPP5A*) gene is suggestively associated with FDG metabolism in the HG (ROIs 1 and 2 from our imaging study; Table 2). Although not much is known about relationship between *INPP5A* and hearing loss, Inpp5a shares protein domains with Synaptojanin 2 (Synj2), both proteins belong to the inositol polyphosphate 5-phosphatase family alongside eight other proteins that remove the 5-position phosphate from phosphoinositides. Phosphoinositides are signaling molecules on cell membranes and essential for normal hearing, and mutant *Mozart* mice (*Synj2*^N538K/N538K^) exhibit progressive hearing loss (Manji, et al., 2011). Mutation in genes affecting phosphoinositide metabolism can be expected to have important consequences in cell function such as disruption in inositol-triphosphate-mediated Ca^2+^ release (Bruzzone and Cohen-Salmon, 2005). Interestingly, disruptions in Ca^2+^ signaling are also to play a role in Alzheimer’s disease (Cheung et al., 2008).

The top SNP rs59570576 in the GWAS with inferior colliculus FDG metabolism is located in an intron of *MIR4300HG,* host gene of microRNA (MIR4300) and some distance downstream of the nearest protein coding gene, *FAM181B* (Family With Sequence Similarity 181 Member B). Mutations in *MIR4300HG* are associated with adolescent idiopathic scoliosis (Ogura et al., 2017) but neither gene has an established role in hearing loss. However, another suggestive SNP, rs10957077 located within an intron of the *TOX* gene (encoding thymocyte selection-associated high mobility group box) is particularly interesting: knockout of this gene in a mouse model *Tox*^tm1b(KOMP)Wtsi^ resulted in a severe hearing loss affecting all frequencies as well as alterations in heart morphology and bone mineral density (https://www.mousephenotype.org/data/genes/MGI:2181659). The inferior colliculus is the main nucleus of the auditory system, receiving information from both ascending and descending sources of the auditory pathway and is the primary source of input to the medial geniculate body within the auditory thalamus, and thus ultimately dictates what information reaches the auditory cortex. Another suggestive-level association of interest is *LRIG3* (leucine rich repeats and immunoglobulin like domains 3) (rs2124517; P-value = 7.7e-06): *LRIG* genes, including *LRIG3,* play an important role during inner ear morphogenesis (Del Rio et al., 2013).

In our imaging results, cluster sizes for effects in the left Heschl’s gyrus were always larger than the right Heschl’s gyrus. Moreover, only the right cochlear nucleus was affected. Similar to visual processing, auditory signals from the ear travel to the auditory cortex mostly contralaterally as majority of fibers taking a contralateral pathway from each ear. Age-related hearing loss is defined as bilateral, progressive hearing loss. Therefore, the asymmetry in hypometabolism in HL cannot be a direct result from unilateral peripheral lack of input to the right cochlear nucleus. Asymmetric differences in microanatomy and volume between left and right Heschl’s gyrus have been noted previously and likely relate to subtle differences in function, although these distinct functions are not yet well defined (Morosan et al., 2001, Rosemann and Thiel, 2019, Warrier et al., 2009, Yao et al., 2015). Our findings suggest that there might be a bigger effect on the left Heschl’s gyrus in ARHL which is responsible for language processing and learning (Norman-haignere et al., 2013, Wong et al., 2008).

The small effect sizes observed for voxel-wise analysis are similar to those detected in previous and smaller studies (Verger et al., 2017, Speck et al., 2020). This may imply that the effect of hearing loss on the brain are small in general or, alternatively, are consistently underestimated, due to the need for age-adjustment during the statistical analysis. Therefore, more work is needed (e.g., using large age-matched cohorts) to properly delineate effects of aging and gearing loss on the brain.

Genetic correlation analyses using GWAS summary statistics revealed that genetics of bilateral Heschl’s gyrus hypometabolism were not correlated with genetics of hearing difficulty in background noise or hearing aid use identified from previous GWAS. However, the direction of the effect was as expected: both traits were negatively correlated with glucose metabolism in HG. In addition, the genes identified related to these GWAS are not shared. This is likely because this GWAS on central auditory pathway structures reflects central processing of sound specifically, whereas the hearing aid and self-reported hearing difficulty GWAS (Wells et al., 2019) reflect both peripheral inputs as well as central processing of sound.

Some limitations of our study include the relatively small sample size for GWAS which may have resulted in a lack of ability to detect genome-wide significant associations with glucose uptake in the HG, IC and CN regions. Secondly, objective pure tone audiometry might provide better criteria for identifying damage to the inner ear and assessing quality of hearing instead of self-reported hearing problems and hearing aid(s) use. However, self-reported hearing data has been used successfully to identify ARHL genes in GWAS (Wells et al., 2019) and it has enabled us to investigate HL in a very large imaging cohort. Thirdly, structural changes in grey matter may contribute to the observed differences in glucose metabolism. This may require a dedicated investigation. Further, there is big age difference between HL and non-HL subjects in this cohort, which may conflate aging effects and disease effects. However, we implemented different age-correction strategies resulting in similar FDG hypometabolism clusters and found that the most conservative approach best captured the auditory processing pathway. Lastly, due to the lack of available replication cohorts, there is no replication study possible for these GWAS.

In conclusion, our study has identified that FDG metabolic decline in structures along the auditory pathway such as Heschl’s gyri, inferior colliculus, and cochlear nucleus are associated with hearing loss. Our GWAS findings have identified a number of candidate genes that might influence FDG uptake in these regions. However, the specific biological pathway(s) underlying the role of these genes in FDG hypometabolism in auditory pathway requires further investigation.

## 5 Data and code availability statement

The data (FDG-PET images) used in the present study were downloaded from the Alzheimer’s Disease Neuroimaging Initiative (ADNI) database (http://adni.loni.usc.edu/). As such, the investigators within the ADNI contributed to the design and implementation of ADNI and/or provided data but did not participate in the analysis or writing of this report. A complete listing of ADNI investigators is available at www.loni.ucla.edu/ADNI/Collaboration/ADNI_Authorship_list.pdf. The toolboxes for imaging data processing and analysis include the Statistical Parametric Mapping (SPM12, https://www.fil.ion.ucl.ac.uk/spm/software/spm12/), FSL (https://fsl.fmrib.ox.ac.uk/fsl/fslwiki/FSL) and FSLEYES v0.26.1 (https://fsl.fmrib.ox.ac.uk/fsl/fslwiki/FSLeyes). The genome-wide association study (GWAS) analysis was done using plink 2.0 (https://www.cog-genomics.org/plink/2.0/). Manhattan plot and identification of lead SNPs were done using Functional Mapping and annotation of genome-wide association studies (FUMA, https://fuma.ctglab.nl/). Genetic correlations were calculated using LD score (LDSC v1.0.1, https://github.com/bulik/ldsc).

## CRediT authorship contribution statement

**Fatin N. Zainul Abidin:** Conceptualization, Methodology, Formal analysis, Writing - original draft, Visualization, Resources. **Marzia A. Scelsi:** Resources, Data curation. **Sally J. Dawson:** Conceptualization, Writing - review & editing, Validation, Supervision, Project administration, Funding acquisition. **Andre Altmann:** Conceptualization, Methodology, Writing - review & editing, Validation, Supervision, Project administration, Funding acquisition.

## Declaration of Competing Interest

The authors declare that they have no known competing financial interests or personal relationships that could have appeared to influence the work reported in this paper.

## References

[b0005] Boyen, K., Langers, D. R. M., Kleine, E. De & Dijk, P. Van. 2013. Gray matter in the brain : Differences associated with tinnitus and hearing loss. Hear. Res. 295, 67–78. 10.1016/j.heares.2012.02.010 22446179

[b0010] Bruzzone R., Cohen-Salmon M. (2005). Hearing the messenger: Ins(1,4,5)P3 and deafness. Nat. Cell Biol..

[b0015] Bulik-Sullivan B.K. (2015). LD Score regression distinguishes confounding from polygenicity in genome-wide association studies. Nat. Genet..

[b0020] Chang C.C., Chow C.C., Tellier L.CAM., Vattikuti S., Purcell S.M., Lee J.J. (2015). Second-generation PLINK: rising to the challenge of larger and richer datasets. Gigascience.

[b0030] Chen C.Y., Pollack S., Hunter D.J., Hirschhorn J.N., Kraft P., Price A.L. (2013). Improved ancestry inference using weights from external reference panels. Bioinformatics.

[b0035] Cheung K.-H., Shineman D., Müller M., Cárdenas C., Mei L., Yang J., Tomita T., Iwatsubo T., Lee V.-Y., Foskett J.K. (2008). Mechanism of Ca2+ disruption in Alzheimer’s disease by presenilin regulation of InsP3 receptor channel gating. Neuron.

[b0040] Cooper E.R.A. (1948). The Development of the Human Auditory Pathway From the Cochlear Ganglion to the Medial Geniculate Body. Cells Tissues Organs.

[b0120] Cruickshanks K.J., Wiley T.L., Tweed T.S., Klein B.E., Klein R., Mares-Perlman J.A., Nondahl D.M. (1998). Prevalence of hearing loss in older adults in Beaver Dam, Wisconsin: The epidemiology of hearing loss study. Am. J. Epidemiol..

[b0045] Cunningham L.L., Tucci D.L. (2017). Hearing Loss in Adults. N. Engl. J. Med..

[b0050] Del Rio T., Nishitani A.M., Yu W.-M., Goodrich L.V. (2013). In vivo analysis of Lrig genes reveals redundant and independent functions in the inner ear. PLoS Genet..

[b0055] Delaneau O., Zagury J.-F., Marchini J. (2013). Improved whole-chromosome phasing for disease and population genetic studies. Nat. Methods.

[b0060] Dukart J., Schroeter M.L., Mueller K., Alzheimer's Disease Neuroimaging Initiative (2011). Age correction in dementia–matching to a healthy brain. PloS One.

[b0065] Eckert M.A., Cute S.L., Vaden K.I., Kuchinsky S.E., Dubno J.R. (2012). Auditory Cortex Signs of Age-Related Hearing Loss. J. Assoc. Res. Otolaryngol..

[b0180] Ferguson M.A., Henshaw H. (2015). Auditory training can improve working memory, attention, and communication in adverse conditions for adults with hearing loss. Front. Psychol..

[b0070] Friston K.J. (1994). Statistical parametric maps in functional imaging: A general linear approach. Hum. Brain Mapp..

[b0075] Gates G.A., Mills J.H. (2005). Presbycusis. Lancet (London, England).

[b0080] Griffiths T.D., Uppenkamp S., Johnsrude I., Josephs O., Patterson R.D. (2001). Encoding of the temporal regularity of sound in the human brainstem. Nat. Neurosci..

[b0085] Han J.H., Lee H.J., Kang H., Oh S.H., Lee D.S. (2019). Brain plasticity can predict the cochlear implant outcome in adult-onset deafness. Front. Hum. Neurosci..

[b9000] Jack C.R. (2008). The Alzheimer's disease neuroimaging initiative (ADNI): MRI methods. Journal of Magnetic Resonance Imaging: An Official Journal of the International Society for Magnetic Resonance in Medicine.

[b0090] Jagust W.J. (2010). The Alzheimer’s Disease Neuroimaging Initiative positron emission tomography core. Alzheimers. Dement..

[b0095] Lee J.S. (2003). PET Evidence of Neuroplasticity in Adult Auditory Cortex of Postlingual Deafness. J. Nucl. Med..

[b0100] Liberman M.C. (2017). Noise-induced and age-related hearing loss: new perspectives and potential therapies. F1000Research.

[b0105] Lin F.R. (2011). Hearing Loss and Incident Dementia. Arch. Neurol..

[b0110] Livingston G., Huntley J., Sommerlad A., Ames D. (2020). Dementia prevention, intervention, and care: 2020 report of the Lancet Commission. Lancet (London, England).

[b0115] Manji S.S.M. (2011). A mutation in synaptojanin 2 causes progressive hearing loss in the ENU-mutagenised mouse strain Mozart. PLoS One.

[b0125] McCarthy S. (2016). A reference panel of 64,976 haplotypes for genotype imputation. Nat. Genet..

[b0130] Morosan P., Rademacher J., Schleicher A., Amunts K., Schormann T., Zilles K. (2001). Human Primary Auditory Cortex: Cytoarchitectonic Subdivisions and Mapping into a Spatial Reference System. Neuroimage.

[b0135] Norman-Haignere S., Kanwisher N., McDermott J.H. (2013). Cortical pitch regions in humans respond primarily to resolved harmonics and are located in specific tonotopic regions of anterior auditory cortex. Journal of Neuroscience.

[b0140] Ogura Y. (2017). A functional variant in MIR4300HG, the host gene of microRNA MIR4300 is associated with progression of adolescent idiopathic scoliosis. Hum. Mol. Genet..

[b0145] Ouda L., Profant O., Syka J. (2015). Age-related changes in the central auditory system. Cell Tissue Res..

[b0155] Rasmussen J.M. (2012). Empirical derivation of the reference region for computing diagnostic sensitive 18fluorodeoxyglucose ratios in Alzheimer’s disease based on the ADNI sample. Biochim. Biophys. Acta - Mol. Basis Dis..

[b0160] Rosemann S., Thiel C.M. (2019). The effect of age-related hearing loss and listening effort on resting state connectivity. Sci. Rep..

[b0165] Scelsi M.A., Khan R.R., Lorenzi M., Christopher L., Greicius M.D., Schott J.M. (2018). Genetic study of multimodal imaging Alzheimer’s disease progression score implicates novel loci. Brain.

[b0170] Shen Y. (2018). Cognitive Decline, Dementia, Alzheimer’s Disease and Presbycusis: Examination of the Possible Molecular Mechanism. Front. Neurosci..

[b0175] Speck I., Arndt S., Thurow J., Blazhenets G., Aschendorff A., Meyer P.T., Frings L. (2020). 18F-FDG PET Imaging of the Inferior Colliculus in Asymmetric Hearing Loss. J. Nucl. Med.: Off. Publ. Soc. Nucl. Med..

[b0185] Talavage T.M., Edmister W.B., Ledden P.J., Weisskoff R.M. (1999). Quantitative assessment of auditory cortex responses induced by imager acoustic noise. Hum. Brain Mapp..

[b0190] Talavage T.M., Gonzalez-Castillo J., Scott S.K. (2014). Auditory neuroimaging with fMRI and PET. Hear. Res..

[b0025] Verger A., Roman S., Chaudat R.M., Félician O., Ceccaldi M., Didic M., Guedj E. (2017). Changes of metabolism and functional connectivity in late-onset deafness: Evidence from cerebral 18F-FDG-PET. Hear. Res..

[b0195] Wang X. (2016). Alterations in gray matter volume due to unilateral hearing loss. Sci. Rep..

[b0200] Warrier C. (2009). Relating Structure to Function: Heschl's Gyrus and Acoustic Processing. J. Neurosci..

[b0205] Watanabe K., Taskesen E., van Bochoven A., Posthuma D. (2017). Functional mapping and annotation of genetic associations with FUMA. Nat. Commun..

[b0210] Wells H.R.R. (2019). GWAS Identifies 44 Independent Associated Genomic Loci for Self-Reported Adult Hearing Difficulty in UK Biobank. Am. J. Hum. Genet..

[b0215] Wong P.C.M., Warrier C.M., Penhune V.B., Roy A.K., Sadehh A., Parrish T.B., Zatorre R.J. (2008). Volume of Left Heschl’s Gyrus and Linguistic Pitch Learning. Cereb. Cortex.

[b0220] World Health Organization. Addressing the rising prevalence of hearing loss. (2018). Available at: http://www.who.int/pbd/deafness/estimates/en/. (Accessed: 14th June 2019).

[b0225] Xu W. (2019). Age-related hearing loss accelerates cerebrospinal fluid tau levels and brain atrophy: a longitudinal study. Aging (Albany. NY).

[b0230] Yao, Z. et al. 2015. A FDG-PET Study of Metabolic Networks in Apolipoprotein E ε4 Allele Carriers. PLoS One 10, e0132300. 10.1371/journal.pone.0132300 PMC4498596 26161964

[b0235] Yueh B., Shapiro N., MacLean C.H., Shekelle P.G. (2003). Screening and Management of Adult Hearing Loss in Primary CareScientific Review. JAMA.

