# Supplementary material for: Glucose hypometabolism in the Auditory Pathway in Age Related Hearing Loss in the ADNI cohort
Source: Neuroimage Clin. 2021 Sep 21;32:102823. doi: 10.1016/j.nicl.2021.102823 (PMC8503577; doi:10.1016/j.nicl.2021.102823)
Supplement: Supplementary data 1 [file mmc1.docx]

Supplementary Material

**Glucose hypometabolism in the Auditory Pathway in Age Related Hearing Loss in the ADNI cohort**

**Fatin N. Zainul Abidin^1,2^, Marzia A. Scelsi^2^, Sally Dawson^1*^_,_ Andre Altmann^2*^** for the Alzheimer’s Disease Neuroimaging Initiative**

1. UCL Ear Institute, University College London, London, UK.
2. Centre for Medical Image Computing, Department of Medical Physics and Biomedical Engineering, University College London, London, UK.

* Joint senior author

** Data used in preparation of this article were obtained from the Alzheimer’s Disease Neuroimaging Initiative (ADNI) database (adni.loni.usc.edu). As such, the investigators within the ADNI contributed to the design and implementation of ADNI and/or provided data but did not participate in analysis or writing of this report. A complete listing of ADNI investigators can be found at: http://adni.loni.usc.edu/wp-content/uploads/how_to_apply/ADNI_Acknowledgement_List.pdf

**Corresponding author:**

Centre for Medical Image Computing (CMIC)
Department of Medical Physics and Biomedical Engineering,
University College London, London, UK.

Tel: +44 20 3549 5631

E-mail address: a.altmann@ucl.ac.uk (A. Altmann)

**Supplementary Figures**


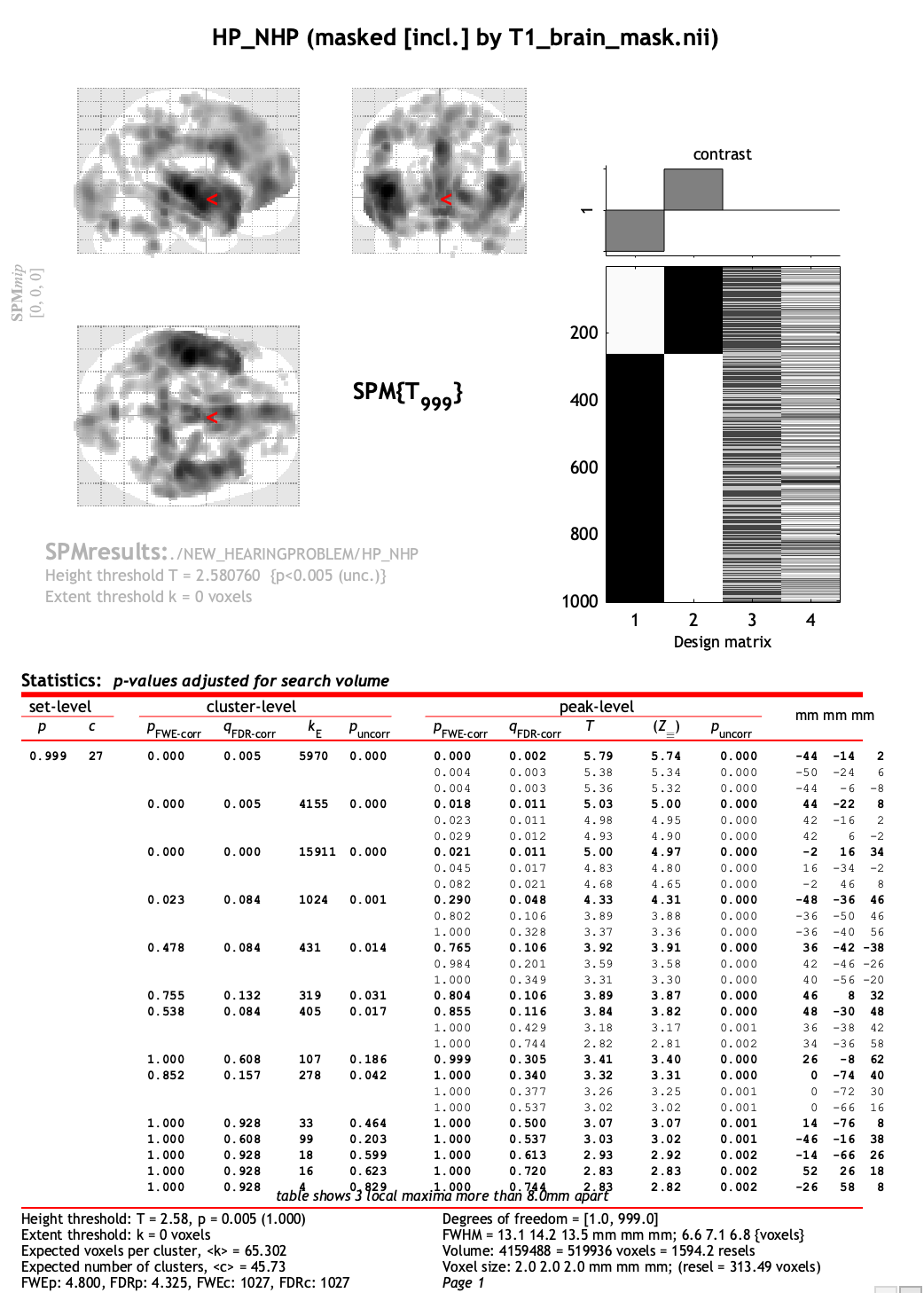


**Figure S1:** SPM output of the areas of lower FDG uptake in participants with hearing loss compared to controls corrected for sex, and years of education only (i.e., no form of age adjustment). The ‘glass brain’ view is shown on the top, and a summary of the major clusters is displayed at the bottom. Voxel-wise statistics are displayed at cluster-level and peak-level. Cluster and peak-level information are displayed for user defined cluster-forming height threshold p-value = 0.005 (uncorrected for multiple testing).


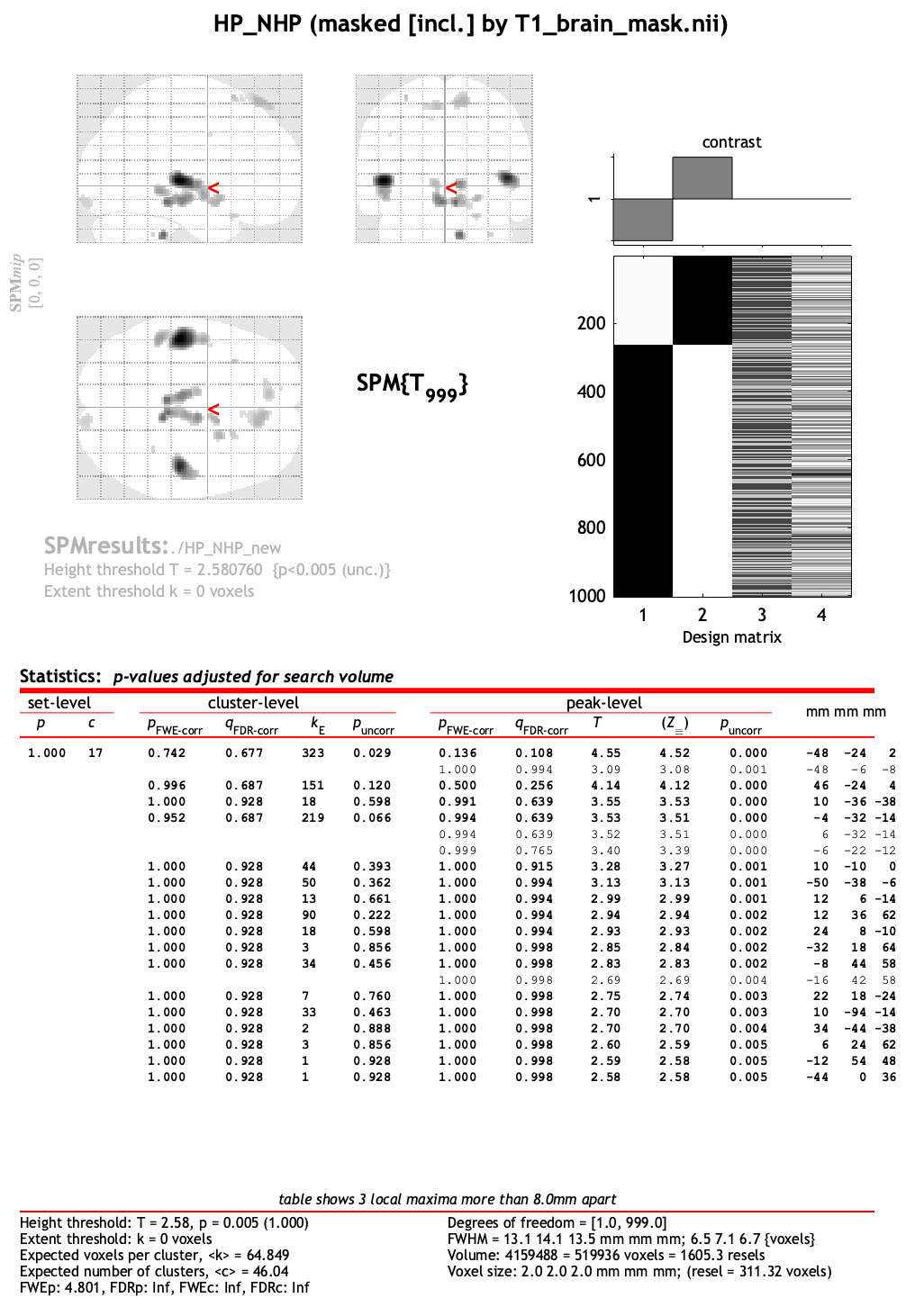


**Figure S2:** SPM output of the areas of lower FDG uptake in participants with hearing loss compared to controls with adjustment of age prior to test and further corrected for sex, and years of education. The ‘glass brain’ view is shown on the top, and a summary of the major clusters is displayed at the bottom. Voxel-wise statistics are displayed at cluster-level and peak-level. Cluster and peak-level information are displayed for user defined cluster-forming height threshold p-value = 0.005 (uncorrected for multiple testing).


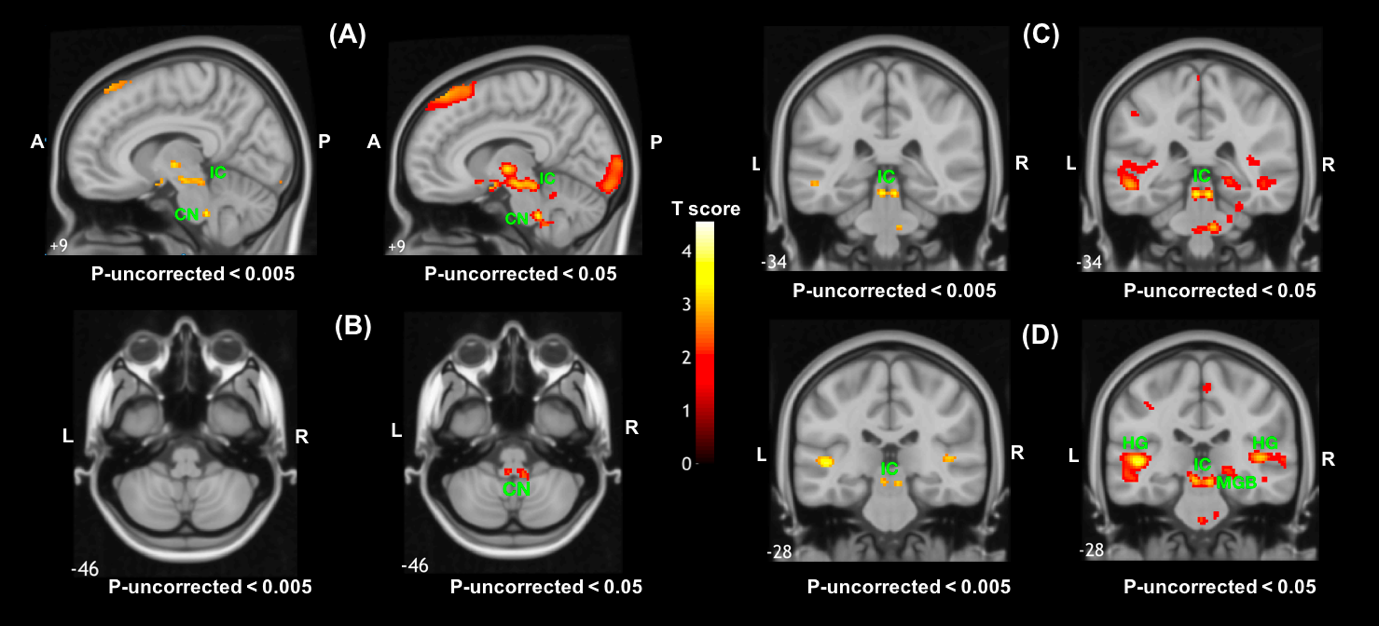


**Figure S3:** Glucose hypometabolism patterns in auditory pathway**.** Data are from participants with hearing loss compared to controls with adjustment of age prior to test and further corrected for sex, and years of education. **(A)** Sagittal section at x = 9 mm showing hypometabolism in the right hemisphere cochlear nucleus (CN) and inferior colliculus (IC) **(B)**, axial section at z = -46 mm showing bilateral hypometabolism of cochlear nuclei **(C)**, coronal section at y = -34 mm showing hypometabolism of inferior colliculus **(D)**, coronal section at y = -28 mm showing hypometabolism of right medial geniculate bodies (MGB) and bilateral Heschl’s Gyrus (HG). All sections are represented at two different cluster-forming height thresholds (p-value = 0.005 and 0.05 uncorrected for multiple testing). Colour scale gives Student's *t* statistic for the comparison between HL and non-HL.


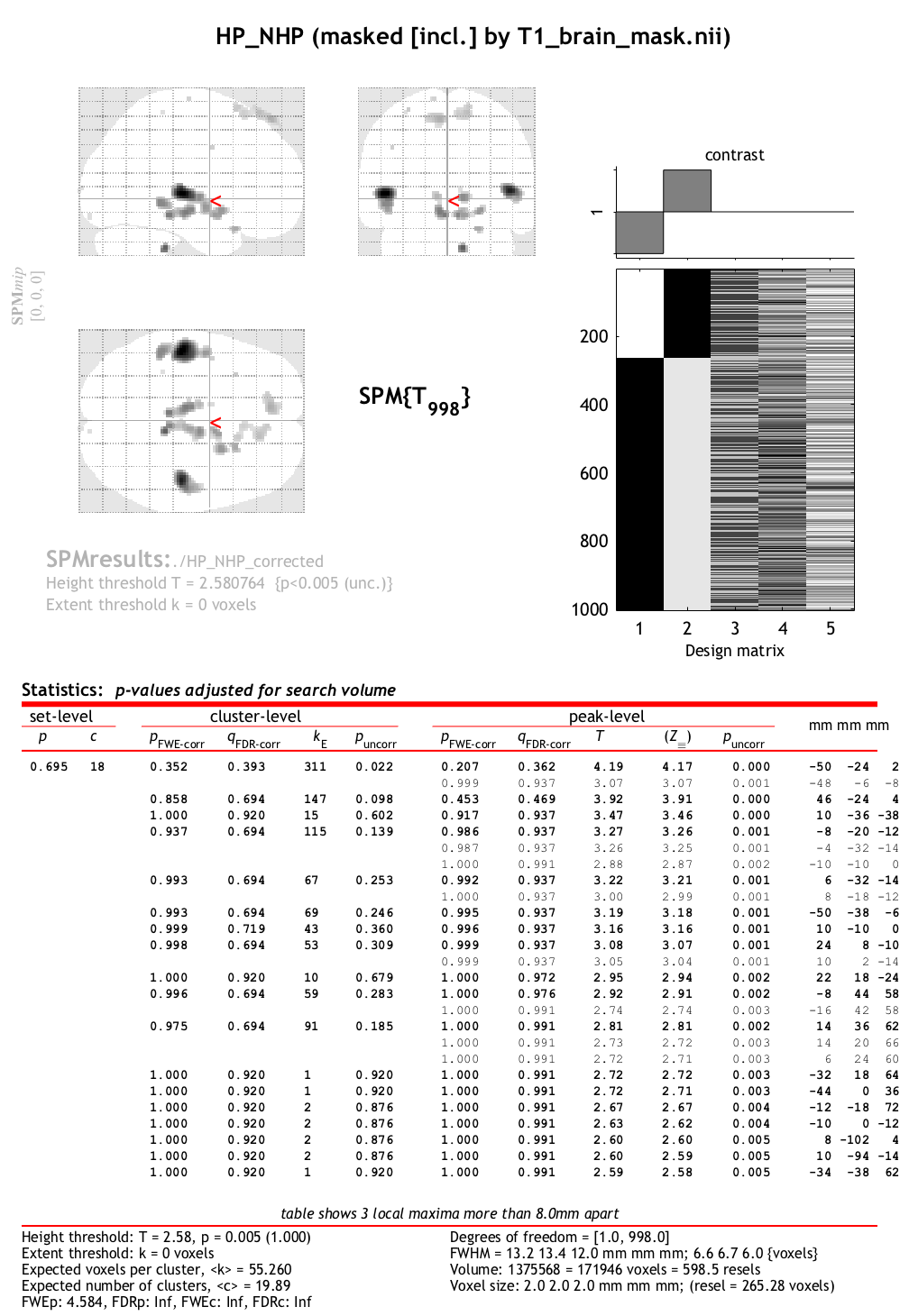


**Figure S4:** SPM output of the areas of lower FDG uptake in participants with hearing loss compared to controls corrected for age, sex, and years of education. The ‘glass brain’ view is shown on the top, and a summary of the major clusters is displayed at the bottom. Voxel-wise statistics are displayed at cluster-level and peak-level. Cluster and peak-level information are displayed for user defined cluster-forming height threshold p-value = 0.005 (uncorrected for multiple testing).


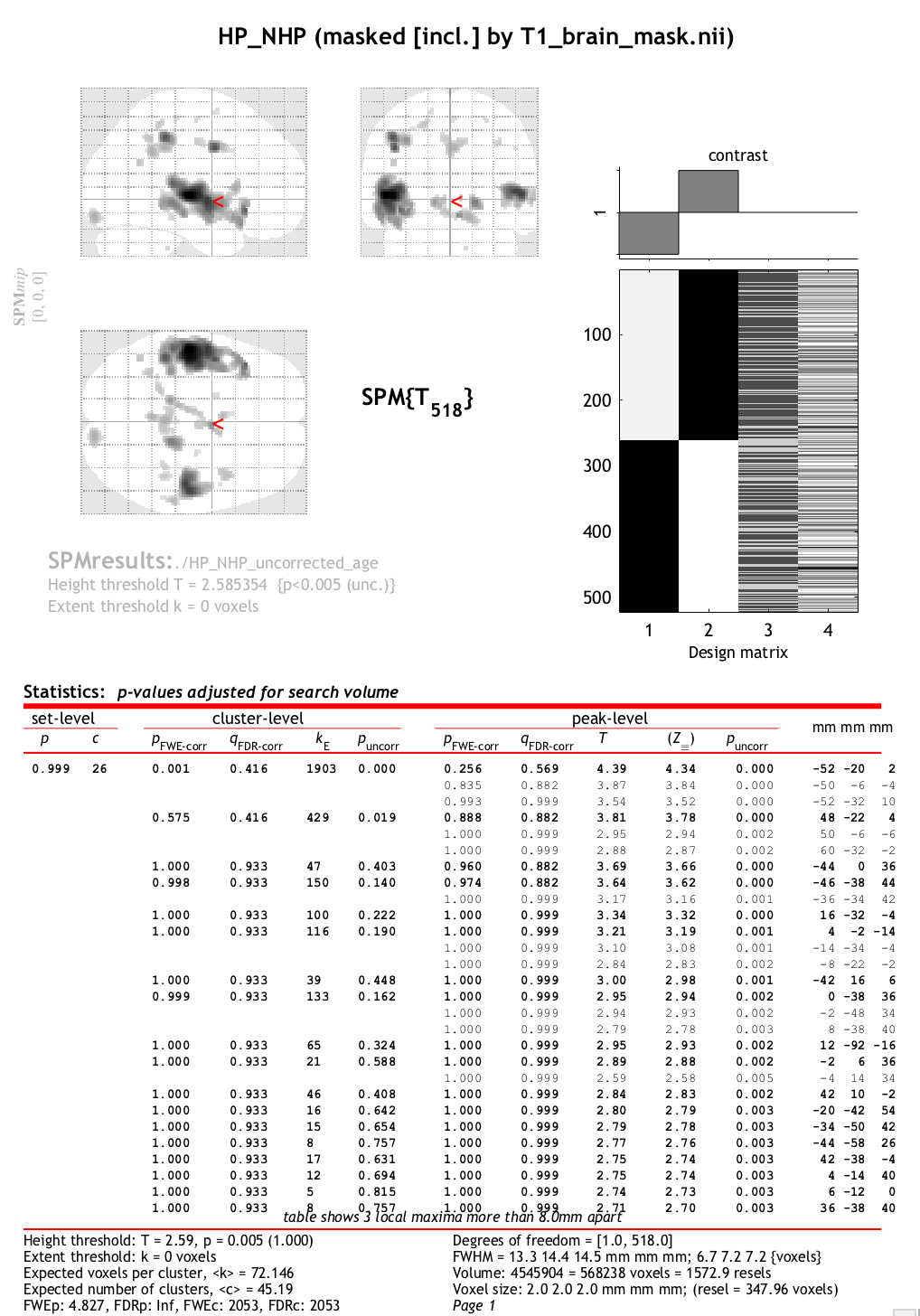


**Figure S5:** SPM output of the areas of lower FDG uptake in participants with hearing loss compared to age-matched controls corrected for sex and years of education only. The ‘glass brain’ view is shown on the top, and a summary of the major clusters is displayed at the bottom. Voxel-wise statistics are displayed at cluster-level and peak-level. Cluster and peak-level information are displayed for user defined cluster-forming height threshold p-value = 0.005 (uncorrected for multiple testing).


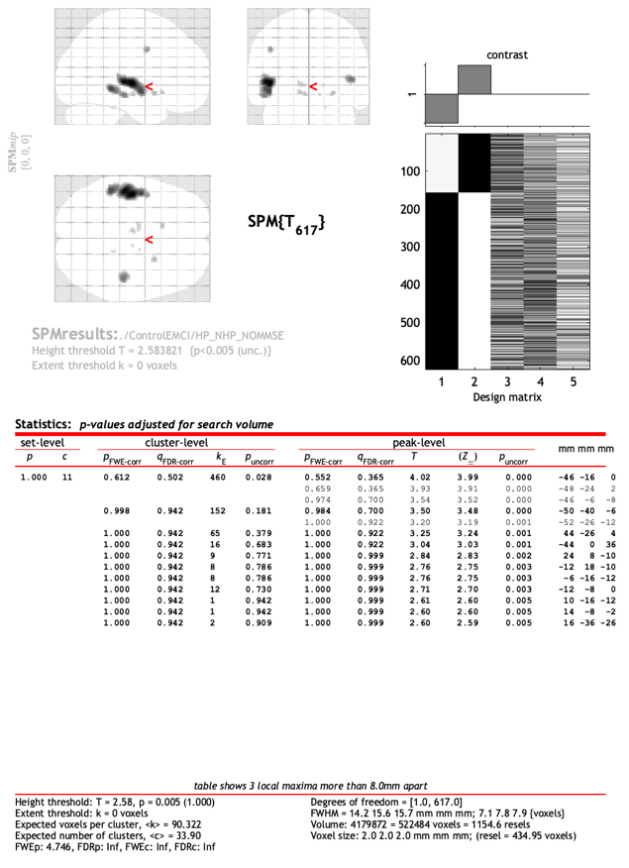


**Figure S6:** SPM output of the areas of lower FDG uptake in participants with hearing loss compared to controls corrected for age, sex, and years of education. Using only subjects with normal cognition or early MCI diagnosis resulting in 156 HL and 466 non-HL subjects. The ‘glass brain’ view is shown on the top, and a summary of the major clusters is displayed at the bottom. Voxel-wise statistics are displayed at cluster-level and peak-level. Cluster and peak-level information are displayed for user defined cluster-forming height threshold p-value = 0.005 (uncorrected for multiple testing).


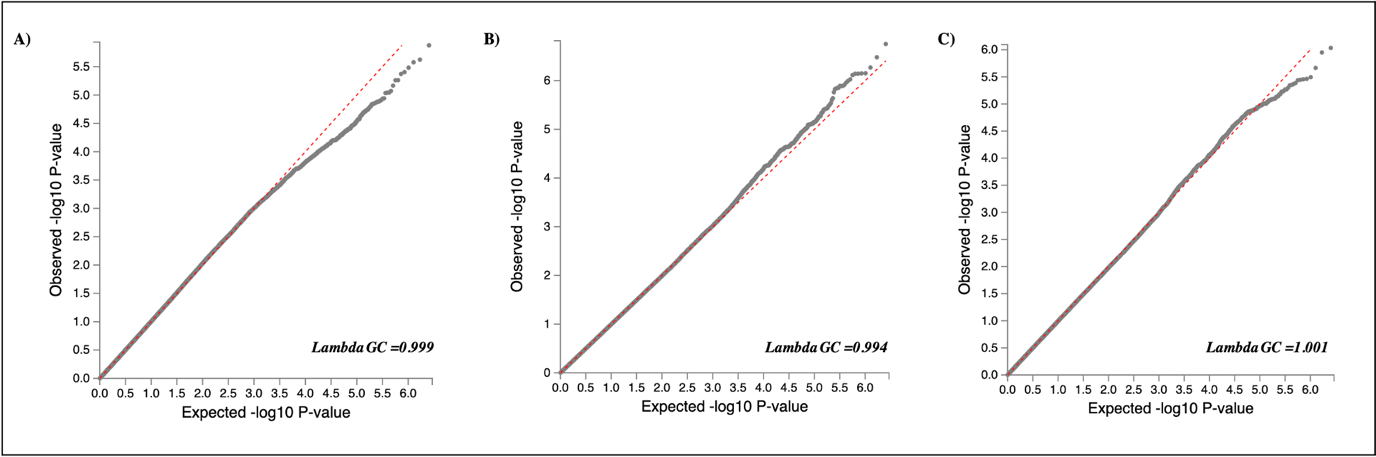
**Figure S7:** The Q-Q plots for **(A)** bilateral Heschl’s gyri/ROI 1&2 GWAS **(B)** inferior colliculus/ROI 3 GWAS **(C)** right cochlear nucleus/ROI4 GWAS of averaged SUVRs within ROIs. The inflation factors, for the GWAS are 0.999, 0.994, 1.001 for (A), (B) and (C) respectively.
